# The identification of disease-induced biomarkers in the urine of BSE infected cattle

**DOI:** 10.1186/1477-5956-6-23

**Published:** 2008-09-05

**Authors:** Sharon LR Simon, Lise Lamoureux, Margot Plews, Michael Stobart, Jillian LeMaistre, Ute Ziegler, Catherine Graham, Stefanie Czub, Martin Groschup, J David Knox

**Affiliations:** 1 Prion Diseases Program, Public Health Agency of Canada, Winnipeg, R3E 3P6, Canada; 2 Department of Medical Microbiology, University of Manitoba, Winnipeg, R3E 0W3, Canada; 3 Department of Pharmacology, University of Manitoba, Winnipeg, R2H 2A6, Canada; 4 Institute for Novel and Emerging Infectious Diseases at the Friedrich-Loeffler Institut, 17493 Greifswald-Insel Riems, Germany; 5 Animal Diseases Research Institute, Canadian Food Inspection Agency, Lethbridge, T1J 3Z4, Canada

## Abstract

**Background:**

The bovine spongiform encephalopathy (BSE) epidemic and the emergence of a new human variant of Creutzfeldt-Jakob Disease (vCJD) have led to profound changes in the production and trade of agricultural goods. The rapid tests currently approved for BSE monitoring in slaughtered cattle are all based on the detection of the disease related isoform of the prion protein, PrP^d^, in brain tissue and consequently are only suitable for post-mortem diagnosis. Objectives: In instances such as assessing the health of breeding stock for export purposes where post-mortem testing is not an option, there is a demand for an ante-mortem test based on a matrix or body fluid that would permit easy access and repeated sampling. Urine and urine based analyses would meet these requirements.

**Results:**

Two dimensional differential gel eletrophoresis (2D-DIGE) and mass spectrometry analyses were used to identify proteins exhibiting differential abundance in the urine of BSE infected cattle and age matched controls over the course of the disease. Multivariate analyses of protein expression data identified a single protein able to discriminate, with 100% accuracy, control from infected samples. In addition, a subset of proteins were able to predict with 85% ± 13.2 accuracy the time post infection that the samples were collected.

**Conclusion:**

These results suggest that in principle it is possible to identify biomarkers in urine useful in the diagnosis, prognosis and monitoring of disease progression of transmissible spongiform encephalopathy diseases (TSEs).

## Background

Bovine Spongiform Encephalopathy (BSE) was first described in the UK in 1985 and became an epidemic that peaked with 37, 280 cases reported in 1992[1]. BSE and other Transmissible Spongiform Encephalopathy (TSE) diseases are untreatable, uniformly fatal degenerative syndromes of the central nervous system (CNS). A post-mortem examination revealing characteristic deposits of an insoluble host encoded protein, astrocytosis and spongiosis is required for definitive diagnosis. The characteristic protein deposits are formed by the accumulation of misfolded isoforms of a host-encoded protein, PrP^c^, or prion protein. The disease associated isoforms are derived from the host protein, PrP^c^, by a posttranslational process and are often distinguished by their partial resistance to proteinase K digestion[2]. The term PrP^d ^is used to denote the presence of abnormal accumulations or isoforms of PrP detected by any method without prejudice as to its biochemical properties, its infectivity, source or host range[3].

The new human variant of Creutzfeldt-Jakob Disease (vCJD) identified in 1996 is thought to have been caused by dietary exposure to BSE infected products[4]. In contrast to typical cases of classical CJD, vCJD seems to affect predominantly young adults. Risk reduction measures implemented in response to BSE and the emergence of this new disease led to profound changes in the production and trade of agricultural goods. To minimize the risk of disease transmission to consumers specified risk materials (SRM), constituting tissues known to harbour high levels of infectivity such as the brain and spinal cord, have been removed from the food chain. In addition, the testing of risk animals and of all slaughtered animals above the age of thirty months for BSE is a requisite for access to many potential markets for beef products. The rapid tests currently approved for BSE monitoring in slaughtered cattle are all based on the detection of the disease related isoform of the prion protein, PrP^d^, in brain tissue and consequently are only suitable for post-mortem diagnosis.

A reliable ante-mortem test would provide an alternative to the routine culling of herds when a confirmed case of BSE is detected. More importantly, in instances such as assessing the health of breeding stock where post-mortem testing is not an option, there is a demand for ante-mortem tests based on a matrix or body fluid that would permit repeated sampling. The development of such assays, based on the detection of PrP^d^, have been complicated by the extremely low amounts of PrP^d ^present in accessible tissues, or in body fluids such as cerebrospinal fluid (CSF), blood and urine [5-8]. Furthermore, the demonstration that most infectivity is associated with protease sensitive forms of PrP^d ^also calls into question the reliability of tests reliant on the association of prion infectivity with the presence of a proteinase K resistant fragment that is measured by Western blotting, enzyme-linked immunosorbent assay, or immunohistochemistry[9].

The advent of the protein misfolding cyclic amplification assay (PMCA), offered a possible solution to this problem[10]. Indeed, the use of modified PMCA assays using relatively defined components has resulted in the detection of PrP^d ^in the CSF, serum and urine of terminal stage hamsters. However, PMCA based methods require further investigation and validation before they are ready for routine use [11-15]. Thus, the identification of alternative biomarkers in accessible tissues or body fluids applicable to the development of diagnostic tests remains a relevant approach.

Urine, due to its ease of collection and comparatively less complex protein profile, is perhaps the ideal matrix for surveillance provided a sufficiently sensitive and specific alternative biomarker for disease can be identified. Previously, it was demonstrated that the presence of protease resistant light chain immunoglobulin in urine may constitute a surrogate marker for prion diseases[6,16]. Building on these results we have used two dimensional differential gel electrophoresis (2D-DIGE) and mass spectrometry analyses to demonstrate that the relative abundance of other proteins in the urine of BSE infected cattle and age matched controls change over the course of the disease. These analyses, performed on biological replicates, identified a single protein able to discriminate between control and infected cattle throughout the course of the disease as well as a subset of proteins able to accurately identify the collection date of the samples. The results indicate that biomarkers in urine may be useful in the diagnosis, prognosis and monitoring of disease progression of transmissible spongiform encephalopathy diseases (TSEs).

## Methods

### Urine

Urine was collected from 4 Simmental cross-breed calves that were orally infected with BSE and 4 age matched controls at 8 month intervals throughout the course of the disease. The calves were infected at 4 months of age. All cattle were scored every second month for clinical signs. BSE was confirmed by immunohistochemistry of the obex[17]. Urine samples were frozen immediately after collection and stored at -80°C until processing. This generated 4 infected and 4 control biological replicates at each of the 6 time points. One sample from this set was not obtained resulting in 47 samples in total.

Urine samples (80–90 mL) were thawed overnight at 4°C. Insoluble particles were removed by a pre-spin, 4°C at 415 g for 5 minutes. The soluble fraction was concentrated with a 5 K MWCO Centricon Plus-70 centrifugal filter unit (Millipore) in a swinging bucket rotor at 4°C and 3400 g for approximately 20 minutes or until volumes reached less than 4 mL. The urine was further concentrated with an Amicon 4 ml 5 K MWCO centrifugal filter unit (Millipore) at 4°C and 7000 g until volumes reached 200 μl.

The concentrated urine samples were purified using a 2D Clean Up kit (GE Healthcare) according to the manufacturer's recommendations. The resulting protein pellets were resuspended in 100 μL of Rehydration Buffer (0.03 M Tris, 8 M Urea, 2 M Thiourea, 2% Chaps, pH 8.5). Samples were adjusted to pH8.5 with the addition of 1–5 μl of 0.05 M NaOH. The concentration of each sample was determined using a 2D Quant kit (GE Healthcare) according to the manufacturer's recommendations. The pooled internal standard was created by combining 100 μg of each sample.

### 2D Gel Electrophoresis

CyDye™ (GE Healthcare) minimal labeling was performed as per the manufacturer's recommendations (400 pmol: 50 μg) with the Cy2 label reserved for the pooled sample. The control and infected samples were labeled in a randomized manner with either Cy3 or Cy5. An equal volume of 2× Rehydration Buffer (0.03 M Tris, 8 M Urea, 2 M Thiourea, 2% Chaps, pH 8.5, 4 mg/ml Dithiothreitol (DTT), 1% IPG buffer pH 4–7) was added to a mixture comprised of 30 μg each of the labeled Pooled, Control and Infected samples. After a 10 minute incubation on ice approximately 400 μl of 1× Rehydration Buffer (0.03 M Tris, 8 M Urea, 2 M Thiourea, 2% Chaps, pH 8.5, 2 mg/ml DTT, 0.5% IPG buffer pH 4–7) and 5 μl of 1% Bromophenol blue (10 mM TrisCl pH 8.5) were added to bring the volume up to a total of 450 μl. The labelled samples were loaded onto a reswelling tray and overlaid with a 24 cm Immobiline DryStrip pH 4–7 (GE Healthcare) and DryStrip Cover Fluid and allowed to rehydrate overnight at room temperature. The strip was transferred to a Manifold filled with 108 mL of DryStrip Cover Fluid and placed on an Ettan IPGphor3 focusing apparatus that was programmed as follows: Step 30 V 8 hrs, Step 500 V 1 hr, Step 1000 V 1 hr, Grad 10000 V 3 hrs and Step 10000 V 3 hrs. A final focusing program: Grad 10000 V 0:20 hr and Step 10000 V until the volt hours reached a total of 55000 was added as required.

Completed isoelectric focusing (IEF) runs were stored at -80°C until the second dimension was run. IEF strips were prepared for second dimension gels by incubating in two different Equilibration Buffer solutions (50 mM Tris-Cl pH8.8, 6 M Urea, 30% Glycerol, 2% SDS, 0.2% Bromophenol Blue, supplemented with either 65 mM DTT – 1^st ^incubation or 135 mM Iodoacetamide – 2^nd ^incubation) for 15 minutes each with gentle rocking.

The prepared IEF strips were placed on 15–20% gradient gels between low fluorescent glass plates (NextGen Sciences). After sealing in place with a 1% agarose solution, the gels were placed in the Ettan DALT6 unit (GE Healthcare) and run at 2 W overnight and then at 100 W until a total of 3100 Vhr was reached.

### Data Acquisition and Analysis

Two randomly selected samples labeled with Cy5 and Cy3 were co-resolved with a Cy2 labeled pooled internal standard on each of 24 gels. Gels were scanned within 24 hours of being run on a Molecular Dynamics Typhoon 9400. Gel images were cropped using Molecular Dynamics Image Quant 5.2 software.

Upon visual inspection of the 24 gels, proteins on three gels were observed to not be well resolved. These three gels were immediately rerun using the same Cy2 labelled standard to obtain gel images suitable for analysis. Acquired gel images were first analyzed in the DeCyder™ Differential In-gel Analysis (DIA) module of the GE HealthCare DeCyder™ 2D Software version 6.5. The DIA generated identical spot feature detection patterns on all images derived from the same gel. This ensured that the internal standard and the sample spot features had identical spot boundaries. Quantification of spot features was achieved by normalizing spot feature volumes against the internal standard.

The DIA files were imported into the Biological Variation Analysis (BVA) module to match spot feature migration patterns and normalize abundance values using the unique signal of each spot feature from the pooled internal standard. The standardized abundance was derived from the normalized spot volume, standardized against the intra gel standard. To obtain a normal distribution around zero the spot feature standardized log abundance values were used for inter-gel spot comparisons.

Multivariate analysis was performed in the DeCyder™ Extended Data Analysis (EDA) module version 1.0. Marker selection and classifier creation were performed using partial least squares for the searching and ranking of spot features and K-nearest neighbor (KNN) to evaluate the spot feature set found.

### Protein Digest

Spot features of interest were manually excised using a Gilson P1000 Pipetman from SYPRO Ruby stained preparative gels and stored in 1% acetic acid. The ART pipet tips were cut with a razor blade to increase the pore size. The gel slices were washed a total of five times; first with sterile water, secondly with 25 mM ammonium bicarbonate and finally three consecutive washes with 25 mM ammonium bicarbonate/50% acetonitrile solution with the last wash being an overnight incubation at 10°C. Gel slices were dehydrated with 100% acetonitrile before adding trypsin (Trypsin Gold, Promega) at 20 μg/ml (in 40 mM ammonium biocarbonate/10% acetonitrile solution) and incubated at 37°C overnight. Tryptic peptides were extracted from the gel slices by washing with 0.1% formic acid and then 0.1% formic acid/50% acetonitrile solutions. The tryptic peptide extracts were vacuum-dried and reconstituted with 10 μl of 5% acetonitrile and 0.1% formic acid.

### LC/MS/MS

Nanoflow LC of tryptic peptide samples was performed with an Agilent 1100 nanoflow LC system equipped with a C_18 _pre-column (Zorbax 300SB-C18, 5 μm, 5 mm × 0.3 mm, Agilent) and a C_18 _analytical column (Zorbax 300SB-C18, 3.5 μm, 15 cm × 75 μm, Agilent). The aqueous mobile phase (solution A) contained 5% acetonitrile and 0.1% formic acid, and the organic mobile phase (solution B) contained 95% acetonitrile and 0.1% formic acid. Samples (5-μl injected) were loaded and washed on the pre-column for 5 minutes with solution A at 50 μl/min. Peptides were then eluted off the pre-column and through the analytical column with a 50 minute profile at 300 nl/min: 1 to 30% solution B over 30 min, 40% to 95% B over 5 minutes, 95% B over 5 minutes, and re-equilibrated for 10 minutes at initial conditions. Peptides were eluted directly into a QStar XL via a nanospray ion source (Applied Biosystems). The ion source was equipped with a 50-μm inner-diameter, fuse-silica needle with a 15-μm tip (PicoTip Emitter, New Objective). Data dependant acquisition was performed with a 10 second cycle: 1-second interval for acquiring intact peptide signal (MS), and three 3-second intervals for collision induced dissociation of the 3 most intense peptide signals in the initial 1-second interval (MS/MS). The MS *m/z *range was 350 to 1500, and the MS/MS *m/z *range was 70 to 2000. Collision energy was automatically determined by the data acquisition software (Analyst QS 1.1). MS/MS data were acquired for the entire LC run.

### Data analysis

Mascot (version 2.2, Matrix Science) search engine was used to search the NCBI database with the MS/MS data. The search parameters were as follows: taxonomy was unrestricted, protein molecular weight was unrestricted, fixed modification was Carbamidomethyl (C), variable modification was Oxidation (M), peptide and fragment mass tolerance was ± 0.3 Da, and up to one missed cleavage was allowed. Individual ion scores > 54 indicate identity or extensive homology (p < 0.05).

## Results

### Gel image acquisition

Urine was collected from 4 Simmental cattle orally infected with BSE and 4 age matched controls at 8 month intervals over the first 40 months of the disease. This generated 4 infected and 4 control biological replicates at each of the 6 time points. An internal gel standard was created by pooling equivalent amounts of protein from each sample. In total 27 gels, each comprised of the internal standard and two biological samples, were run to obtain gel images suitable for analysis. Ultimately, 46 gel images representing 46 biological samples and 24 gel images of the internal standard were used for analyses (Table 1).

**Table 1:** Sample loading and labelling matrix.

**group**	**cow**	**gel no.**	**dye**	**mpi**	**group**	**cow**	**gel no.**	**dye**	**mpi**
infected	38	na	na	0	control	67	23	Cy3	0
infected	40	1	Cy5	0	control	69	22	Cy5	0
infected	53	15	Cy3	0	control	72	11	Cy5	0
infected	54	17	Cy5	0	control	73	13	Cy3	0

infected	38	24	Cy3	8	control	67	1	Cy3	8
infected	40	8	Cy3	8	control	69	3	Cy5	8
infected	53	13	Cy5	8	control	72	23	Cy5	8
infected	54	8	Cy5	8	control	73	9	Cy3	8

infected	38	19	Cy5	16	control	67	24	Cy5	16
infected	40	27	Cy3	16	control	69	14	Cy3	16
infected	53	25	Cy3	16	control	72	16	Cy5	16
infected	54	18	Cy3	16	control	73	6	Cy5	16

infected	38	19	Cy3	24	control	67	15	Cy5	24
infected	40	21	Cy3	24	control	69	17	Cy3	24
infected	53	na	na	24	control	72	3	Cy3	24
infected	54	26	Cy5	24	control	73	21	Cy5	24

infected	38	10	Cy5	32	control	67	14	Cy5	32
infected	40	12	Cy3	32	control	69	9	Cy5	32
infected	53	25	Cy5	32	control	72	2	Cy5	32
infected	54	6	Cy3	32	control	73	16	Cy3	32

infected	38	10	Cy3	40	control	67	20	Cy3	40
infected	40	11	Cy3	40	control	69	2	Cy3	40
infected	53	18	Cy5	40	control	72	26	Cy3	40
infected	54	12	Cy5	40	control	73	27	Cy5	40

The disease state, cow identity and months post infection (mpi) identify the sample. Dyes and gels were randomly assigned to the 4 biological replicates of infected and control cows to minimize the influence of dye bias and gel to gel variation. The 2 infected samples marked NA were either not collected (cow 52, 24 mpi) or no suitable gel image was obtained (cow 38, 0 mpi)

**Figure 1:**
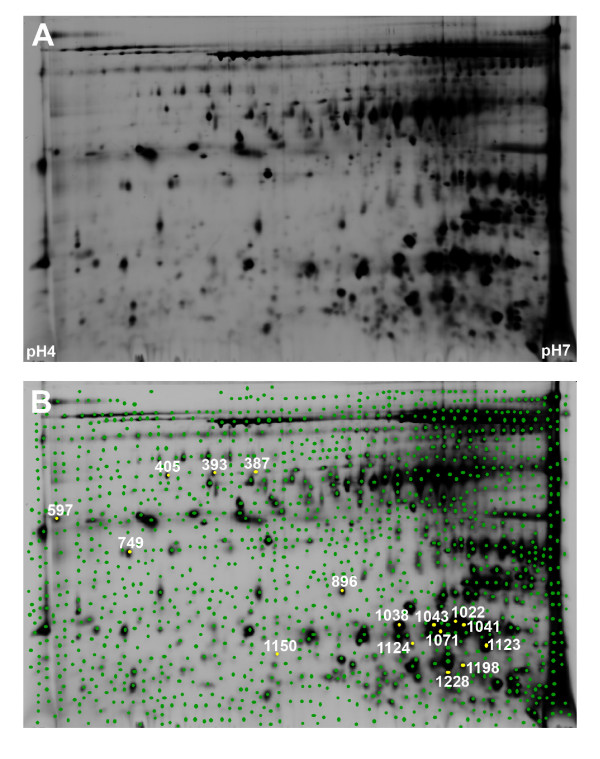
**Representative Cy2-labelled internal standard proteome gel image illustrating proteins resolved in the pH4-7 range**. The gel image as loaded into the DIA module prior to spot detection (A). In panel B the 1329 spot features, including spots at the edges of the gel that were outside the pH range of the 1^st ^dimension separation, are each denoted by a green dot. The position of the 16 spot features used in the class prediction classifier have been marked with yellow and the associated master gel spot feature number assigned to the same spot features on all gels by the DeCyder BVA module are shown.

The acquired gel images were analyzed using the DeCyder™ DIA and BVA software modules. Manual landmarking of 12 spot features across 24 gel images of the internal standard was performed in order to improve the accuracy of the gel-to-gel matching process. This resulted in the detection, quantification, and matching of 1329 master spot features across the 24 gels, all containing the same internal standard (Figure 1).

### PCA analyses

Multivariate analyses of protein expression data derived from the BVA were performed using the DeCyder™ Extended Data Analysis Software (EDA). The gel images were first grouped such that the 6 samples from each individual animal formed a group. This resulted in 8 groups each representing one of 8 biological replicates. The data were filtered so that only the 36 spot features exhibiting statistically significant (ANOVA p < 0.01) changes in abundance and present on all 46 gel images were considered in the following analyses.

**Figure 2:**
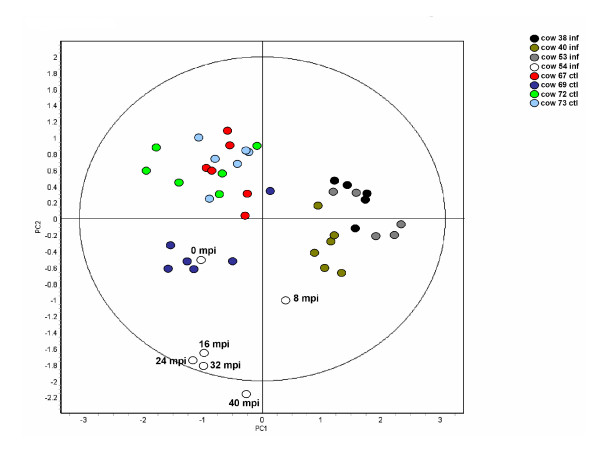
**Principle component analysis of the 8 biological replicates**. The samples obtained from individual infected and control cows clustered together indicating that disease is the factor that most influences the differential abundance observed in the urine samples. The time of the 6 sample collections from cow #54 are given to illustrate that the urine proteome of this animal diverged further from all the other animals as the disease progressed. This analysis was based on the 36 spot features exhibiting statistically significant (ANOVA p < 0.01) changes in abundance and present on all 46 gel images. (PC1 = 36.3, PC2 = 15.2).

Principle component analysis (PCA) on this filtered data set was performed to identify the relative contributions of the inherent differences between individuals and disease state on the variance exhibited by the 8 biological replicates. The PCA analysis demonstrated that the cows generally segregated into infected and control groups indicating that the disease status of the animals was the primary factor affecting the differential abundance of urinary proteins (Figure 2). The exception was cow #54. At no time point did this animal cluster with the other infected animals and it further diverged from all other animals as the disease progressed.

The reason for the atypical pattern demonstrated by this animal is not known, but it is interesting to note that it had an atypical phenotype as well. Cow #54 developed clinical signs, as determined by regular scoring for clinical signs (20 minutes/animal), between 40–46 months post infection (mpi), but then phenotypically recovered before reaching the terminal stage of the disease at 56 mpi. In contrast, cows #38 and #53 reached the terminal stage of the disease at 44 mpi and cow #40 at 48 mpi. The behaviour of cow #54 was atypical, not only with respect to the other three infected cattle considered in this study, but from the other 10 infected cows in the herd that were allowed to reach the terminal stage of the disease. For the purposes of this experiment all the data representing this animal were excluded from subsequent analyses. BSE infection was confirmed in all four cases by immunohistochemical analyses of the obex.

The 40 remaining gel images were then grouped according to disease state and months post infection. Control and infected cows at each time point formed a group. This resulted in 10 groups each representing either the 3 remaining infected cows or the 4 control cows at a particular point in the experiment. Samples collected from these 7 cows prior to the start of the experiment formed an 11th group (normal). The data were again filtered revealing 56 spot features that exhibited statistically significant (ANOVA p < 0.01) changes in abundance and were present on all 40 gel images for consideration in the following analyses.

**Figure 3:**
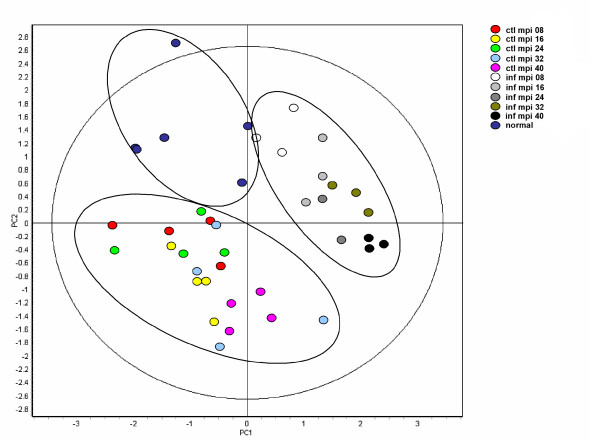
**Principle component analysis of different disease states followed throughout the disease progression**. Ellipses have been drawn to illustrate the clustering of the 3 groups (BSE infected, control and normal). Within the infected group it can also be seen that the individual time points cluster together. A somewhat similar but less pronounced pattern is observed in the control samples. This analysis is based on the 56 spot features exhibiting statistically significant (ANOVA p < 0.01) changes in abundance and present on all 40 gel images. (PC1 = 38.6, PC2 = 23.0).

PCA analysis on this filtered data set was performed to identify the relative contributions of time and disease state on the variance observed (Figure 3). The cows again segregated into infected and control groups further indicating that the disease status of the animals was the primary cause of the differential abundance in urinary proteins observed. Nonetheless, within the infected group it was also observed that the individual time points clustered together and generally moved down and to the right as disease progressed. A somewhat similar, but less pronounced pattern was observed in the control samples. This indicates that time did factor into the differential abundance of urinary proteins observed. That time played a role in the urinary protein profile was reinforced by the observation that the animals at 0 mpi formed a distinct group (normal).

**Figure 4:**
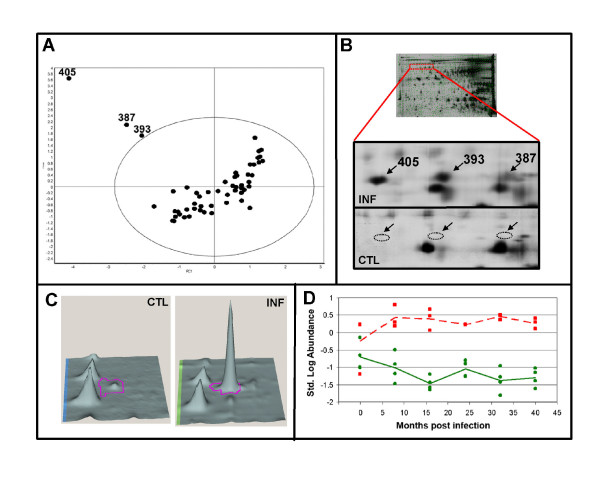
**Differentially abundant spot features**. Principle component analysis of 56 spot features that exhibited statistically significant (ANOVA p < 0.01) changes in abundance and were present on all 40 gels (A). The red rectangle on the gel image shows the region on the gels where the potential outliers were situated. Two magnified views of this region showing infected and control images (B). 3D images of spot feature 405 showing the 23.68 fold increase in abundance observed at 8 mpi (C). Graphical representation of the standardized log abundance data obtained for spot feature 405 (D).

PCA of the 56 spot features that exhibited statistically significant (ANOVA p < 0.01) changes in abundance and were present on all 40 gels revealed 3 outliers representing either mismatched features or strongly differentially abundant features (Figure 4A). Visual inspections of all potential outliers were made in the corresponding BVA file containing the gel images. The 3 spot features clearly observed in the infected image were not visible in the control image of the gel (Figure 4B). Visualization of the shape of the peak representing spot feature 405 and those in the surrounding area demonstrated that the matches were legitimate (Figure 4C). The increase in abundance of 405 ranged from 17 to 77 fold over the course of the experiment. Graphical representation of the standardized log abundance obtained for the spot feature 405 demonstrated strong differential abundance of the spot feature throughout the experiment. Note the segregation of the control and infected biological replicates at each time point (Figure 4D).

### Classifier creation

In order to determine whether the differences suggested by the PCA analyses were sufficient to discriminate control from infected animals the marker selection and classifier creation functions of the EDA module were used. Initially this was performed on the pooled data obtained from all control, infected and normal samples. The data, as before, were filtered so that only spot features exhibiting statistically significant (ANOVA p < 0.01) changes in abundance and present on all 40 gel images were considered.

The partial least squares method was used for the searching and ranking of spot features and K-nearest neighbor (KNN) was used to evaluate the set of biomarkers found. The use of 16 biomarkers and KNN classification demonstrated that the training data set could be classified with 83.3% ± 18.3 accuracy (Table 2). Removal of the confounding normal samples led to the identification of a single spot feature (405) that could discriminate between the remaining control and infected sample sets with 100% accuracy.

**Table 2:** Classification Matrix

83.33% ± 18.3	Class Prediction		
	control	infected	normal

control	**20**	0	3
infected	0	**14**	0
normal	0	0	**3**
no class	0	0	0
error	0	0	3

The classifier created was applied to the training set to assign gel maps with respect to disease state. The classification matrix shows an overview of the classification of the gel maps. Gels that were correctly classified are displayed in bold type. A classifier containing 16 biomarkers was used to discriminate between the 3 groups with 83.33% ± 18.3 accuracy. A single protein was able to discriminate between control and infected samples with 100% accuracy.

In order to evaluate the information concerning disease progression contained in the urine samples the 14 gel images of the infected samples were considered separately. The data were filtered so that only the 25 spot features exhibiting statistically significant (ANOVA p < 0.01) changes in abundance and present on all 14 gel images were considered. The classifier created using the 16 spot features identified by marker selection was able to classify the time post infection that the infected samples were collected with 85% ± 13.2 accuracy. The two misclassified samples were placed into the immediately proceeding time point (Table 3).

**Table 3:** Disease Progression Matrix

85% ± 13.2	Infected Progression				
	08 mpi	16 mpi	24 mpi	32 mpi	40 mpi

08 mpi	**3**	0	0	0	0
16 mpi	0	**3**	0	0	0
24 mpi	0	0	**2**	2	0
32 mpi	0	0	0	**1**	0
40 mpi	0	0	0	0	**3**
no class	0	0	0	0	0
error	0	0	0	2	0

The classifier created was applied to the training set to assign gel maps with respect to disease progression. The classification matrix shows an overview of the classification of the gel maps. Gels that were correctly classified are displayed in bold type. A classifier containing 16 biomarkers was used to discriminate between the 5 time points with 85% ± 13.2 accuracy. The two misclassified samples at 32 mpi were placed into the immediately proceeding sampling time.

In order to parse out those changes due to disease progression from those associated with aging a similar analysis was performed on the control samples. The classifier created using 16 of the spot features identified by marker selection was able to classify with 85% ± 19.1 accuracy the time post infection that the control samples were collected (Table 4). Two of the three misclassified samples were placed into the immediately proceeding time point.

**Table 4:** Aging Matrix

85% ± 19.1	Control Progression				
	08 mpi	16 mpi	24 mpi	32 mpi	40 mpi

08 mpi	**4**	1	0	0	0
16 mpi	0	**3**	1	1	0
24 mpi	0	0	**3**	0	0
32 mpi	0	0	0	**3**	0
40 mpi	0	0	0	0	**4**
no class	0	0	0	0	0
error	0	1	1	1	0

The classifier created was applied to the training set to assign gel maps with respect to sample collection time. The classification matrix shows an overview of the classification of the gel maps. Gels that were correctly classified are displayed in bold type. A classifier containing 16 biomarkers was used to discriminate between the 5 time points with 85% ± 19.2 accuracy. The misclassified samples at 16 and 24 mpi were placed into the immediately proceeding sampling times. The misclassified sample at 32 mpi was classified as 16 mpi.

**Figure 5:**
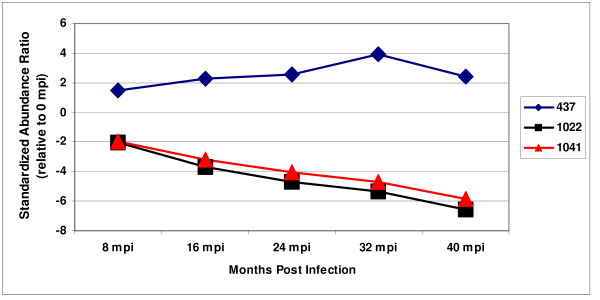
**Proteins that exhibited a steady increase or decrease in abundance throughout disease progression**. The average standardized abundance ratios of the top three ranked proteins used in the disease progression classier (437, 1041, 1022) are shown. The consistent increase or decrease in abundance over the course of the experiment illustrates the utility of the relative abundance of these spot features in classifying the urine samples with respect to date post infection.

The 16 spot features used by the 3 classifiers are provided in Table 5. The rank assigned to the spot features denotes the relative contribution of each protein to the classification. The standardized abundance ratio of the top three ranked proteins in the disease progression classifier were plotted with respect to time post infection (Figure 5). The steady increase or decrease in abundance observed over the course of the experiment illustrated the utility of the relative abundance of the spot features in classifying the urine samples with respect to date post infection. Three of the spot features utilized to predict disease progression (597, 1022, 1041) were also used in disease class prediction indicating that these markers are disease specific. Another disease progression spot feature (710) also appeared among the control progression markers indicating that changes in the abundance of this spot feature were age related.

**Table 5:** Biomarker sets used to create classifiers.

	Control Progression		Disease Progression		Class Prediction	
	Spot #	Rank	Spot #	Rank	Spot #	Rank

1	161	10	125	9	**387**	2
2	168	8	127	6	393	3
3	395	2	239	4	405	1
4	473	6	297	10	*597*	11
5	482	6	437	1	749	12
6	603	3	*597*	5	896	10
7	608	5	626	6	*1022*	8
8	*710*	8	*710*	11	1038	7
9	860	10	740	8	**1041**	12
10	1006	10	841	9	1043	4
11	1007	4	911	7	1071	12
12	1101	7	*1022*	3	1123	9
13	1127	9	*1041*	2	1124	5
14	1200	1	1078	12	**1150**	10
15	1278	4	1103	11	1198	8
16	1457	9	1318	10	1228	6

In this particular instance each classifier was composed of 16 biomarkers. Biomarkers that appear in more than one classifier are in italics. The three biomarkers in the class prediction set that were not identified by MS analysis (387,1041,1150) are in bold faced type.

### LC/MS/MS analyses

The 16 biomarkers making up the class prediction classifier (Figure 1B), designed to discriminate between control, infected and normal samples, were excised from a preparative gel and subjected to protein identification using mass spectrometry and database interrogation as described in Materials and Methods. MS analysis identified 5 unique proteins, not including redundancies likely due to post-translational modifications or proteolysis, and enabled proteins to be assigned to 13 of the 16 spot features. A summary of the results of this analysis are shown in Table 6. See Additional File 1 for additional protein statistics.

**Table 6:** Thirteen of the 16 spot features included in the class prediction classifier were identified. The average ratios at each time point are given.

		Average Ratio (infected/control)					
Spot	Protein ID	0 mpi	8 mpi	16 mpi	24 mpi	32 mpi	40 mpi

387		2.04	12.35	10.91	7.63	2.46	8.70
393	clusterin (Bos Taurus)	2.44	11.36	9.17	5.23	10.12	6.76
405	clusterin (Bos Taurus)	4.05	23.68	77.54	17.00	54.36	33.80
597	Ig Gamma-2 chain C region (Bos taurus)	1.98	-1.4	3.58	2.35	3.03	4.93
749	simlar to GCAP-11/uroguanylin (Bos taurus)	-1.33	1.80	1.03	-1.23	1.03	1.15
896	cystatin E/M (Bos Taurus)	-1.15	1.13	-1.09	1.23	-1.33	1.10
1022	cathelicidin antimicrobial peptide (Bos Taurus)	-1.10	-1.12	-1.02	-2.54	-1.61	-1.91
1038	cathelicidin 1 (Bos Taurus)	-3.84	1.03	-1.91	-4.13	-2.46	-1.87
1041		-1.10	-1.17	-1.14	-2.77	-1.80	-2.18
1043	cathelicidin 1 (Bos Taurus)	1.35	1.27	1.13	-2.99	-1.61	-1.60
1071	cathelicidin 1 (Bos Taurus)	1.23	-1.47	-1.20	-2.29	-1.76	-1.81
1123	simlar to GCAP-11/uroguanylin (Bos taurus)	1.35	-1.14	1.05	-2.98	-1.91	-2.51
1124	simlar to GCAP-11/uroguanylin (Bos taurus)	1.27	-3.52	-2.25	-3.19	-2.28	-2.55
1150		1.38	-3.02	-1.66	-1.08	-5.36	1.01
1198	simlar to GCAP-11/uroguanylin (Bos taurus)	-1.34	-3.00	-1.49	-1.04	-4.16	-1.43
1228	simlar to GCAP-11/uroguanylin (Bos taurus)	-1.11	-5.84	-5.19	-6.95	-3.20	-3.98

The 5 different proteins representing the 13 spot features were: clusterin, Ig Gamma-2 chain C region, similar to GCAP-11/uroguanylin, cystatin E/M, and cathelicidin1. Some of the redundancies appeared to be due to post-translational modifications that created charge-related isoforms that had different iso-electric points, but indistinguishable molecular masses. For example, 2 spot features identified as clusterin (393, 405), appeared to meet this criteria (Figure 4B). It is also interesting to note that the differential abundance of one of the isoforms is much more robust than the other (Figure 4A).

## Discussion

Recent advances in 2-dimensional gel electrophoresis technologies, namely the introduction of fluorescent dyes, which allow multiple samples to be co-separated and visualized on one 2-D gel, have increased the utility of this methodology for the discovery of robust protein biomarkers for disease [18-20]. For example, power analysis for 2D-DIGE has demonstrated statistical power of >0.8 for detecting 2-fold changes at p ≤ 0.01 with 4 biological replicates[21]. In our experiment urine samples representing 4 control and 4 age matched infected cattle were run with an internal standard. The internal standard, created by pooling aliquots of all biological samples in the experiment, was run on all gels within the experiment thereby creating an intrinsic link across all gels. Normalization of the internal standard across gels allowed the ratio of relative abundance of the same protein to be compared directly, separating gel-to-gel variation from biological variation. Differences in protein abundance were then determined by comparing the ratio obtained from one fluorescent labelled sample directly with another.

Changes in the amount of a given soluble protein in urine can result from a change in its concentration in the blood plasma, a change in the function of the glomerular filter, an alteration in the proximal tubule scavenging system or a change in local production and excretion[22]. No proteinuria indicating a change in the glomerular filter was observed in response to BSE infection (Additional file 2). In addition, no significant difference (p < 0.01) in the standardized log of abundance for cystatin was observed at any time point when compared to the corresponding control (Additional file 3). Cystatin is a low molecular weight cysteine proteinase inhibitor that is freely filtered by the renal glomeruli and reabsorbed in the proximal tubules. Consequently cystatin serves as an indicator of the health of the proximal tubule scavenging system[23,24]. The absence of change in cystatin abundance indicated that the proximal tubule scavenging system was unaffected by BSE infection. Together these results suggest that the differential abundances observed in response to BSE infection were likely due to changes in the protein concentration in the blood plasma or a disease associated alteration in local production.

One of the identified classifier proteins that exhibited increased abundance in response to infection was immunoglobulin Gamma-2 chain C region. Increased immunoglobulin has previously been observed in the urine of scrapie infected hamsters and in one report it was speculated that this was the result of a nephritic syndrome [5,6,16]. In contrast, the data presented suggests that the increased abundance of immunoglobulin in urine associated with BSE was probably due to a change in immunoglobulin concentration in the blood plasma. Nonetheless, the detection of differential abundance of another immunoglobulin protein in urine by an unbiased screen lends support to the suggestion that immunoglobulin light chain may constitute a surrogate marker for TSE diseases[6,16].

In addition to immunoglobulin Gamma-2 chain C region and cystatin, the 3 other identified classifier proteins were well known urinary proteins. One was the antimicrobial peptide cathelicidin that is produced in the kidney by the epithelial cells that line the urinary tract. When exposed to bacteria the levels of cathelcidin mRNA are known to rapidly increase, however, even in the absence of microbes the epithelial surface of the urinary tract are continuously bathed with cathelicidin[25,26]. A second protein was the natriuretic peptide uroguanylin that is produced in the small intestine and kidney. In response to salt loading no increase in circulating uroguanylin is observed indicating that the natriuretic effect of uroguanylin is in part mediated by increased renal production that inhibits tubular resorption of ions from the glomerular filtrate[27]. These characteristics suggested that the decreased abundance of both cathelicidin, observed after 24 mpi, and uroguanylin throughout the experiment were probably the result of decreased renal production and excretion. The precise cause of the decreased production and the possible effects of the altered abundance on prion pathobiology are not known.

The third protein, identified as clusterin, was able to distinguish between infected animals and age matched controls with 100% accuracy throughout the experiment. Clusterin is a multifunctional glycoprotein with nearly ubiquitous tissue distribution[28]. Increased abundance of clusterin in association with TSE diseases has been reported previously and has included increased expression in astrocytes as well as a significant accumulation in cerebrospinal fluid and blood plasma[29]. Thus, increased amounts of circulating clusterin may have caused the increased clusterin abundance observed in the urine of the BSE infected animals. Despite the power of clusterin as a biomarker of BSE in this experiment, the increased clusterin abundance in CSF observed in models of other neurodegenerative diseases, such as Alzheimer's disease, and in response to a variety of renal insults raises doubts as to the specificity of clusterin per se as a biomarker of BSE in cattle [30-34]. However, the specificity of the particular isoform of clusterin observed to best discriminate between BSE infected and control cattle remains to be seen.

EDA analyses also demonstrated that the differential abundance of different subsets of proteins provided accurate measures of disease progression and aging. This was an unexpected result, but the ability to follow disease progression by monitoring the differential abundance of a subset of proteins has potential applications as a prognostic indicator or in the assessment of the therapeutic benefit of potential treatments. Furthermore, while markers of disease progression must be sensitive to changes in disease state and present in easily accessible tissues that permit repeated sampling, they do not require the same high disease specificity as diagnostic markers. The ultimate utility of these markers of disease progression will be determined by their identification and validation as well as their applicability to clinically relevant disease models. Significantly, the markers of disease progression demonstrated very little overlap with those identified as able to track age. This indicates that they were a measure of disease specific progression and that their identification may also provide insight into the pathology of these diseases.

The results demonstrate that in principle it is possible to identify biomarkers of TSE disease by analyzing changes in the urine protein profile provoked by the disease. Extending the present study to larger numbers of cattle and to those of other strains will test the value of the biomarkers identified. Even more promising markers may have been missed due to the bias of 2D-DIGE to the identification of abundant proteins. This shortfall will be addressed in future studies by utilizing a variety of pre-fractionation methods.

## Authors' contributions

JDK, MG, SC, CG have made substantial contributions to conception and design of the experiment. SLRS, LL, MP, JL, UZ, MS performed the acquisition of data. JDK carried out the analysis and interpretation of data. JDK, SLRS, MP, LL, SC have been involved in drafting the manuscript or revising it critically for important intellectual content. All authors read and approved the final manuscript.

## Supplementary Material

Additional File 1 Results of LC/MS/MS analyses and protein statistics. Thirteen of the 16 spot features included in the class prediction classifier were identified. For each identified protein the mascot score, the number of peptides, the % coverage and the corresponding NCBI identifier are provided. Individual ions scores > 54 indicate identity or extensive homology (p < 0.05). The average ratios at each time point are also given. Click here for file

Additional file 2 Statistical Analysis of Protein Concentration in the Urine Samples. Protein concentrations of urine are evaluated to determine if there was any difference amongst cows and whether or not the concentration changed throughout the course of the disease. Click here for file

Additional file 3 Statistical Analysis of Cystatin Abundance in Control and Infected Urine samples collected at each of the 5 time points. The relative cystatin abundances found in control and infected urine were compared. Click here for file
