# Supplementary material for: The identification of disease-induced biomarkers in the urine of BSE infected cattle
Source: Proteome Sci. 2008 Sep 5;6:23. doi: 10.1186/1477-5956-6-23 (PMC2546380; doi:10.1186/1477-5956-6-23)
Supplement: Additional File 1 — Results of LC/MS/MS analyses and protein statistics. Thirteen of the 16 spot features included in the class prediction classifier were identified. For each identified protein the mascot score, the number of peptides, the % coverage and the corresponding NCBI identifier are provided. Individual ions scores > 54 indicate identity or extensive homology (p < 0.05). The average ratios at each time point are also given. [file 1477-5956-6-23-S1.doc]

|  | | | | | | Average Ratio (infected/control) | | | | | |  |
| --- | --- | --- | --- | --- | --- | --- | --- | --- | --- | --- | --- | --- |
| Spot | Protein ID | Mascot score | Peptides | Coverage | NCBI ID | 0  mpi | 8  mpi | 16 mpi | 24 mpi | 32 mpi | 40 mpi | ANOVA |
| 387 |  |  |  |  |  | 2.04 | 12.35 | 10.91 | 7.63 | 2.46 | 8.70 | 1.19E-10 |
| 393 | clusterin (Bos Taurus) | 60 | 2 | 5% | gi 27806907 | 2.44 | 11.36 | 9.17 | 5.23 | 10.12 | 6.76 | 4.33E-15 |
| 405 | clusterin (Bos Taurus) | 43 | 1 | 3% | gi 27806907 | 4.05 | 23.68 | 77.54 | 17.00 | 54.36 | 33.80 | 2.89E-15 |
| 597 | Ig Gamma-2 chain C region (Bos taurus) | 64 | 2 | 8% | gi 89611 | 1.98 | -1.4 | 3.58 | 2.35 | 3.03 | 4.93 | 1.44E-03 |
| 749 | simlar to GCAP-11/uroguanylin (Bos taurus) | 62 | 1 | 5% | gi 119890356 | -1.33 | 1.80 | 1.03 | -1.23 | 1.03 | 1.15 | 1.75E-03 |
| 896 | cystatin E/M (Bos Taurus) | 242 | 2 | 40% | gi 61097917 | -1.15 | 1.13 | -1.09 | 1.23 | -1.33 | 1.10 | 3.83E-06 |
| 1022 | cathelicidin antimicrobial peptide (Bos Taurus | 69 | 1 | 7% | gi 27806725 | -1.10 | -1.12 | -1.02 | -2.54 | -1.61 | -1.91 | 1.55E-06 |
| 1038 | cathelicidin 1 (Bos Taurus) | 153 | 2 | 22% | gi 27807341 | -3.84 | 1.03 | -1.91 | -4.13 | -2.46 | -1.87 | 1.43E-03 |
| 1041 |  |  |  |  |  | -1.10 | -1.17 | -1.14 | -2.77 | -1.80 | -2.18 | 6.28E-06 |
| 1043 | cathelicidin 1 (Bos Taurus) | 190 | 2 | 22% | gi 27807341 | 1.35 | 1.27 | 1.13 | -2.99 | -1.61 | -1.60 | 4.68E-04 |
| 1071 | cathelicidin 1 (Bos Taurus) | 177 | 2 | 22% | gi 27807341 | 1.23 | -1.47 | -1.20 | -2.29 | -1.76 | -1.81 | 1.67E-03 |
| 1123 | simlar to GCAP-11/uroguanylin (Bos taurus) | 53 | 1 | 5% | gi 119890356 | 1.35 | -1.14 | 1.05 | -2.98 | -1.91 | -2.51 | 7.23E-05 |
| 1124 | simlar to GCAP-11/uroguanylin (Bos taurus) | 50 | 1 | 5% | gi 119890356 | 1.27 | -3.52 | -2.25 | -3.19 | -2.28 | -2.55 | 2.23E-05 |
| 1150 |  |  |  |  |  | 1.38 | -3.02 | -1.66 | -1.08 | -5.36 | 1.01 | 6.17E-03 |
| 1198 | simlar to GCAP-11/uroguanylin (Bos taurus) | 72 | 1 | 5% | gi 119890356 | -1.34 | -3.00 | -1.49 | -1.04 | -4.16 | -1.43 | 2.02E-03 |
| 1228 | simlar to GCAP-11/uroguanylin (Bos taurus) | 55 | 1 | 5% | gi 119890356 | -1.11 | -5.84 | -5.19 | -6.95 | -3.20 | -3.98 | 1.56E-05 |

**Additional File 1.** Results of LC/MS/MS analyses and protein statistics
