# Supplementary material for: The identification of disease-induced biomarkers in the urine of BSE infected cattle
Source: Proteome Sci. 2008 Sep 5;6:23. doi: 10.1186/1477-5956-6-23 (PMC2546380; doi:10.1186/1477-5956-6-23)
Supplement: Additional file 2 — Statistical Analysis of Protein Concentration in the Urine Samples. Protein concentrations of urine are evaluated to determine if there was any difference amongst cows and whether or not the concentration changed throughout the course of the disease. [file 1477-5956-6-23-S2.doc]

**Additional File 2.** Statistical Analysis of Protein Concentration in the Urine Samples (μg/mL).

|  | MPI | | | | | |  |
| --- | --- | --- | --- | --- | --- | --- | --- |
| Cow ID | 0 | 8 | 16 | 24 | 32 | 40 | Average |
| 67 | 14.19 | 2.36 | 12.71 | 15.79 | 9.10 | 25.29 | 13.24 |
| 69 | 49.34 | 15.29 | 21.00 | 19.31 | 5.14 | 36.11 | 24.37 |
| 72 | 5.93 | 43.30 | 16.39 | 5.41 | 27.78 | 17.89 | 19.45 |
| 73 | 5.68 | 26.07 | 11.69 | 10.43 | 33.39 | 9.21 | 16.08 |
| 38 | 0.98 | 1.13 | 8.65 | 18.69 | 18.48 | 18.22 | 11.03 |
| 40 | 39.38 | 7.64 | 14.13 | 16.97 | 15.52 | 27.29 | 20.16 |
| 53 | 10.34 | 22.82 | 9.14 | na | 11.84 | 17.51 | 14.33 |
| 54 | 5.35 | 1.95 | 7.46 | 12.26 | 4.46 | 1.70 | 5.53 |
| Average | 16.40 | 15.07 | 12.65 | 14.12 | 15.71 | 19.15 |  |

No significant difference observed in the protein concentration of the urine between cows (ANOVA p≥0.0107322)

No significant difference observed in the protein concentration of the urine over time (ANOVA p≥0.921281)
