# Supplementary material for: The identification of disease-induced biomarkers in the urine of BSE infected cattle
Source: Proteome Sci. 2008 Sep 5;6:23. doi: 10.1186/1477-5956-6-23 (PMC2546380; doi:10.1186/1477-5956-6-23)
Supplement: Additional file 3 — Statistical Analysis of Cystatin Abundance in Control and Infected Urine samples collected at each of the 5 time points. The relative cystatin abundances found in control and infected urine were compared. [file 1477-5956-6-23-S3.doc]

**Additional File 3**. Statistical Analysis of Cystatin Abundance in Control and Infected Urine (T-Test, Assuming Equal Variances)

|  |  | **Standardized Log Abundance** | | | | **T-Test** |
| --- | --- | --- | --- | --- | --- | --- |
| mpi 0 | CTL | -1.006466 | -0.689309 | -0.123852 | -0.984077 | P<0.910382 |
|  | INF | -0.357935 | -1.137354 |  |  |  |
| mpi 8 | CTL | -0.378398 | -0.502427 | -0.008600 | -0.089905 | P<0.992237 |
|  | INF | -0.232996 | -0.385606 | -0.120574 |  |  |
| mpi 16 | CTL | -0.252853 | -0.290035 | -0.190332 | -0.127105 | P<0.031327 |
|  | INF | -0.136721 | -0.008600 | 0.012837 |  |  |
| mpi 24 | CTL | -0.243038 | -0.164353 | -0.346353 | -0.505150 | P<0.149198 |
|  | INF | 0.170262 | -0.167317 | -0.161368 |  |  |
| mpi 32 | CTL | -0.354108 | 0.029384 | 0.328380 | -0.096910 | P<0.428737 |
|  | INF | 0.161368 | -0.060698 | -0.127105 |  |  |
| mpi 40 | CTL | 0.037426 | 0.056905 | -0.187521 | 0.056905 | P<0.071246 |
|  | INF | 0.301030 | 0.382017 | 0.110590 |  |  |
